# Acute Thumb Reconstruction with Iliac Crest Bone Graft and Groin Flap: A Case Report

**DOI:** 10.31729/jnma.8264

**Published:** 2023-09-30

**Authors:** Niresh Shrestha, Santosh Batajoo, Sweta Jaiswal, Om Prasad Shrestha

**Affiliations:** 1 Department of Orthopedics, B&B Hospital Pvt. Ltd., Gwarko, Lalitpur, Nepal

**Keywords:** *case reports*, *injury*, *thumb*

## Abstract

Crush injury with bone loss results in shortening the length of the thumb. Most of the immediate intervention is amputation and stump closure. Revision amputation with stump closure gives loss of functional length and the patient is unable to do daily activities as before. In most of all hand functions, the opposition of the thumb plays an important role. Reconstruction of the thumb with iliac crest bone graft with its functional length is one of the major achievements for the patient. As in our case, the patient is right-hand dominant he is more concerned about the functional length of the thumb, whatever the aesthetic appearance. Here we present a case of a 24-year-old male with thumb reconstruction in a severely injured thumb with loss of bone and soft tissue just distal to the base of the proximal phalanx. Nine months postoperatively, the patient showed a great outcome with restored thumb length, function, grip strength, and a good range of motion.

## INTRODUCTION

Complete loss of thumb results in impairment of 40% of the hand, 36% of the upper extremity, and 22% of whole person impairment.^1^ The site of injury addresses surgical options for thumb reconstructions. Middle-third injuries result in loss of pinch and strong grasp.^2^ The aim of reconstruction is to maintain the length of the thumb. The current standard of treatment is, compared to no reconstruction, any of the procedures are beneficial for thumb reconstruction.^3^ This case report describes an osteoplastic thumb reconstruction for near total amputation of thumb just distal to metacarpophalangeal joint with iliac crest bone graft with groin flap with satisfactory functional length and grip strength.

## CASE REPORT

**Figure 1:**
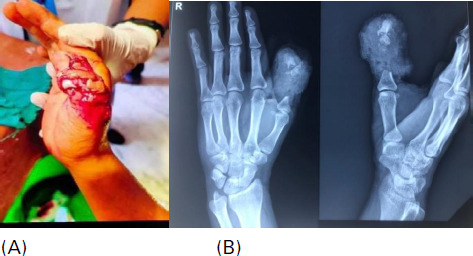
A) The middle third of the right thumb injury to distal to the metacarpophalangeal joint, B) Plain radiograph of thumb at the time of presentation.

A 24-year-old gentleman, right-hand dominant, body mass index (BMI) of 21 kg/m^2^, presented with trauma to the right thumb subtotal amputation due to a saw machine. The patient had concerns about pain, bleeding, and loss of soft tissue and bone in the right thumb. The patient had no co-morbidities, non-smoker, and had no drug allergic history. No family history of any chronic illness. Before presentation to our centre, the patient has treated at another centre and planned for revision amputation and closure but he denies it. On laboratory examination, all the blood parameters are within normal limits. On physical examination, a primary survey was done. On local examination, the right thumb showed an extensive complex injury with segmental loss of the proximal phalanx with a comminuted open fracture of the distal phalanx. The anterior skin bridge was intact with the loss of dorsal soft tissues over distal and proximal phalanx. The tip of the distal phalanx was intact with a nail bed and nail plate (Figure 1).

The capillary refill and sensation of the thumb were intact with the skin bridge. There was a loss of the thumb flexor pollicis longus tendon, and extensor pollicis longus tendon but the metacarpophalangeal joint remains intact. A quick decision was made and planned for operative management exploration and proceed.

**Figure 2:**
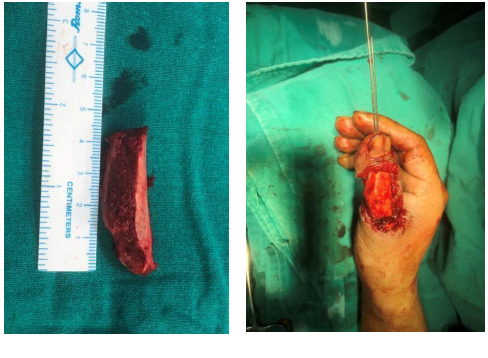
Iliac crest bone graft and fixation of bone graft with K-wire.

**Figure 3:**
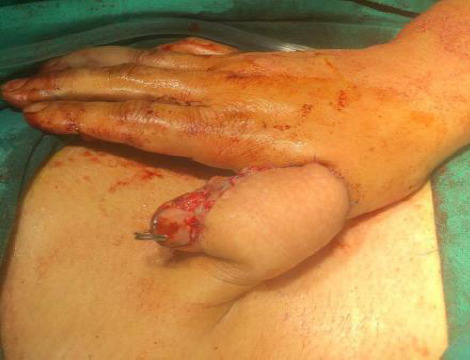
Groin flap with iliac bone graft wrapped.

Primarily a meticulous debridement was done, and wound swabs and tissue cultures were sent. A broad-spectrum prophylactic antibiotic was started which was continued for 5 more days and was followed by analgesic, and proton pump inhibitors. After multiple debridements, there was around a 4 cm long bony defect, tendon laceration with loss of dorsal soft tissue. The distal phalanx tip was intact with a nail bed, nail plate, and pulp. The comminuted bones were removed. The interphalangeal joint was non-salvageable. We found that the best option would be a reconstruction of the thumb with an iliac crest bone graft around 4x1x1 cm. The bone was fixed with 1.2 mm two K-wires and for the soft tissue coverage, the same side groin flap was done (Figures 2 and 3).

**Figure 4:**
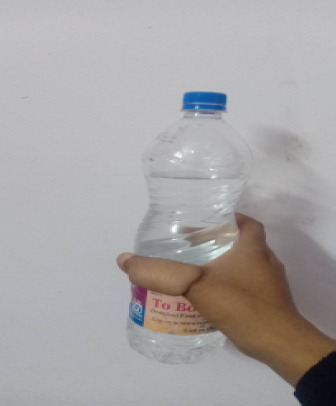
Clinical picture of holding the bottle 9 months postoperatively.

Postoperatively, there was no complication. Regular wound care was done. Three weeks post-operative, the flap detachment procedure was done. After flap detachment the patient underwent physiotherapy range of motion, and opposition exercises are started. Follow-up was done in 4 weeks, 6 weeks, 8 weeks, and 12 weeks. Heavy lifting is avoided for at least 6 weeks. Six weeks after the initial operation, K-wire removal was done and full mobilization was started. The functional outcome was assessed with a Kapandji score for opposition of the thumb.^4^ At 9 months of surgery, the bone was fully consolidated with adequate soft tissue coverage and power was assessed by holding the bottle (Figure 4).

Range of motion in the metacarpophalangeal joint is 0-60 degrees with 8 points on the Kapandji opposition score. The donor site healed without complication.

## DISCUSSION

Here we present a technique in which a severely injured thumb could be salvaged by bone graft and groin flap. Reconstruction of the thumb aims to restore the length of the thumb, stable wound healing, and early active use of the thumb. In this case, the injury of the thumb is the middle third. At this level, the more concern is the functional length of the thumb. Reconstruction is necessary for the patient. Possible alternatives for the patient include bone cement with delayed bone graft, first dorsal metacarpal artery (FDMA) flap, revision amputation, index pollicization, toe to thumb transfer, etc.^5^ Non-vascularized iliac crest bone graft was used to reconstruct the functional length of the thumb, with a 13% risk of graft failure, 33% of reduction of graft width, and 55% reduction in graft length.^6^ There was minimal resorption of the graft appears to achieve a good vascular bed to receive the graft. Iliac crest bone graft surface remodelling will shape to meet the demands of the new biomechanical forces applied to it.^7^ This was seen in our bone graft.

Opposition is an important hallmark of the thumb. Thus the reconstruction with painless skin coverage and acceptable length to enable opposition and circumduction.^8^ The thumb amputations are divided into four functional categories: soft-tissue deficit with acceptable length, subtotal amputation with borderline length, total amputation with preservation of the carpometacarpal joint, and total amputation with the destruction of the carpometacarpal joint.^9^ This classification would help us to decide on reconstruction. Clinical examination and radiographs are used for the determination of the level of loss. Groin flaps are reliable and versatile flaps which required multiple-stage surgery, poor sensation, and absorption of bone graft were the main factor of groin flap and iliac crest bone graft.^10^ Donor site morbidity and the scar were much more acceptable to the patient than the radial forearm flap.^10^

Thumb replantation using microsurgical techniques is always a priority. Successful replantation rates of total and subtotal amputations vary from 55% to 93%. Arterial and venous thrombosis has been considered the most common complications post-replantation. When replantation is not feasible, reconstructive techniques should be considered.

This procedure does not need microsurgical skills, this can be performed in those cases where replantation and revascularization cannot be done. It is a very useful procedure for reconstructing bone and soft tissue preserving thumb functional length after traumatic injury with excellent function and acceptable aesthetic outcomes.
